# Plain Language Summary of Publication: Design of the Phase 3 AZALEA Trial of Nipocalimab in Severe Hemolytic Disease of the Fetus and Newborn

**DOI:** 10.1055/a-2529-4150

**Published:** 2025-03-06

**Authors:** Yosuke Komatsu, Victor Olusajo, Leona E. Ling, Shumyla Saeed-Khawaja, May Lee Tjoa, Trishan Vaikunthanathan, Arpana Mirza, Kenneth J. Moise

**Affiliations:** 1 Janssen Pharmaceutical Companies of Johnson & Johnson, Cambridge, Massachusetts; 2 Dell Medical School, The University of Texas at Austin, Austin, Texas; 3 Comprehensive Fetal Care Center at Dell Children's Medical Center, Austin, Texas

**Keywords:** neonatal Fc receptor blocker, nipocalimab, HDFN, intrauterine transfusion, red blood cell alloimmunization, safety, efficacy, study design

## Abstract

This article is a plain language summary of publication (PLSP) of the following article: Design of a phase 3, global, multicenter, randomized, placebo-controlled, double-blind study of nipocalimab in pregnancies at risk for severe hemolytic disease of the fetus and newborn by Komatsu et al published in *American Journal of Perinatology* on September 17, 2024 (doi:10.1055/a-2404-8089). This PLSP describes the design of the phase 3 AZALEA clinical trial in pregnant participants at risk for developing severe hemolytic disease of the fetus and newborn (HDFN). In this study, researchers will determine if an investigational treatment called nipocalimab can be used safely and effectively to treat pregnant individuals who are at risk for severe HDFN. This PLSP will help members of the public, including individuals and families affected by HDFN, understand the study. It may also be helpful for health care professionals. An infographic summary of this article is available in the Supplementary Material .

Design of a Phase 3, Global, Multicenter, Randomized, Placebo-Controlled, Double-Blind Study of Nipocalimab in Pregnancies at Risk for Severe Hemolytic Disease of the Fetus and Newborn

## Synopsis

### What Is This Summary About?

This is a summary of a publication describing the design of the phase 3 AZALEA clinical trial in pregnant participants at risk for developing severe hemolytic disease of the fetus and newborn (HDFN). In this study, researchers will determine if an investigational treatment called nipocalimab can be used safely and effectively to treat pregnant individuals who are at risk for severe HDFN.

### What Happens in the Study?

The study is enrolling approximately 120 pregnant individuals who are at risk for severe HDFN based on their pregnancy history. They will be recruited by approximately 60 global centers with expertise in maternal–fetal medicine and the treatment of HDFN. As severe HDFN can develop quickly, all participants will have weekly appointments to monitor for fetal anemia until planned delivery at 37 to 38 weeks of pregnancy. In addition, participants are randomly divided in a 2:1 ratio into two groups to receive weekly intravenous infusions of either nipocalimab or placebo (an inactive treatment used as a control group to see whether the observed effects are actually caused by the therapy). Treatment will begin between 13 and 16 weeks of pregnancy and end at 35 weeks of pregnancy. After delivery, maternal participants will be monitored at several follow-up visits for 6 months and infants will be monitored for 2 years.

### What Key Results Will the Study Provide?

The main goal of this study is to measure the ability of nipocalimab treatment to prevent or decrease the most serious effects of severe HDFN and to further understand the side effects of nipocalimab treatment. To measure the effect of nipocalimab treatment on the disease, the proportion (%) of participants whose pregnancies do not have fetal loss, intrauterine transfusion (IUT), fetal hydrops, or neonatal death will be compared between the nipocalimab and placebo groups. To identify side effects that may be caused by nipocalimab, all side effects experienced by maternal participants will be monitored and recorded at weekly visits during pregnancy and at periodic follow-up visits for 6 months after delivery and in their child for the first 2 years of life.

### Who Is This Summary for?

This summary will help members of the public, including individuals and families affected by HDFN, understand the study. It may also be helpful for health care professionals. More detailed information and references can be found in the original article. The link to the original article can be found at the end of this summary.

### Who Sponsored the Study?

This study is sponsored by Janssen Research & Development, LLC, a Johnson & Johnson Company.

## Content

### What Is Hemolytic Disease of the Fetus and Newborn?

**Fig. 1:**
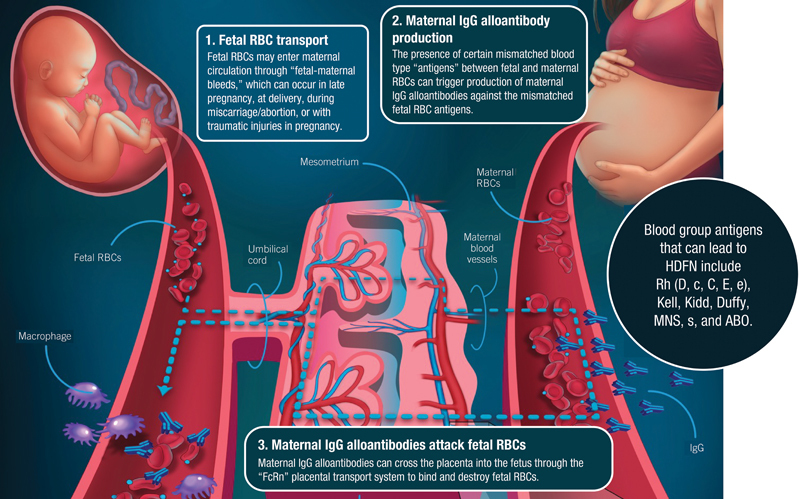
Development of HDFN.

HDFN is a rare disease that can occur in pregnancy when there are features on the fetus' red blood cells (RBCs) that are not present on the maternal RBCs (paternal blood type antigens). Small to moderate placental bleeds or abnormal large bleeds that cause fetal RBCs to enter maternal blood during pregnancy and delivery can trigger the maternal immune system to produce “alloantibodies” against the paternal blood type antigens present on the fetus or newborn. These alloantibodies are mostly of the immunoglobulin G (IgG) type. They are transferred from the maternal blood to fetal blood by the placenta, where they can attack the fetal RBCs causing hemolytic anemia. The anti-RBC IgG alloantibodies that most commonly cause severe HDFN are made against the Rhesus D (RhD), Rhesus c (Rhc), and Kell blood group antigens ( Fig. 1 ).

**Fig. 2:**
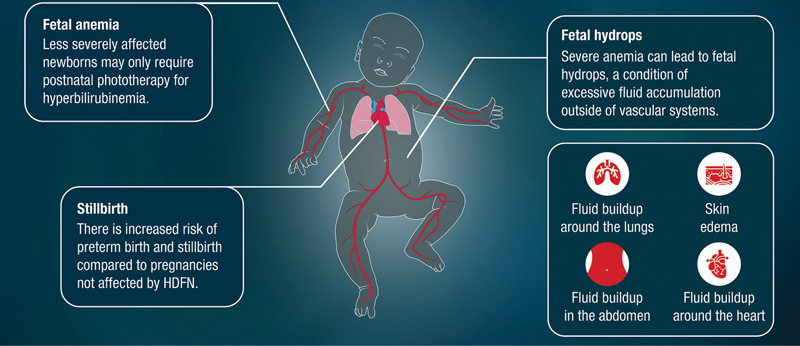
Effects of HDFN on the fetus and neonate.

In milder cases, the newborn or infant may develop jaundice or anemia from the destruction of their RBCs. In severe cases, the fetus develops moderate to severe anemia, which requires timely and sometimes repeated IUTs of RBCs to the fetus during pregnancy to prevent serious consequences for the unborn baby. After birth, these newborns can also experience jaundice, serious hyperbilirubinemia (high levels of bilirubin), and/or anemia requiring exchange transfusion (a procedure where a neonate's blood is removed and replaced with donor blood or plasma to remove alloantibodies and bilirubin from the blood and to treat severe anemia in the neonate), simple transfusion (a procedure where a neonate or infant receives RBCs from a donor to increase the RBC count in the neonate's or infant's blood), and other procedures. Furthermore, there is a high chance (80–90%) that severe HDFN will occur again in future pregnancies if the fetus has the mismatched blood type antigen targeted by the maternal alloantibodies ( Fig. 2 ).

### How Is a Pregnancy at Risk of Severe Hemolytic Disease of the Fetus and Newborn Treated Now?

**Fig. 3:**
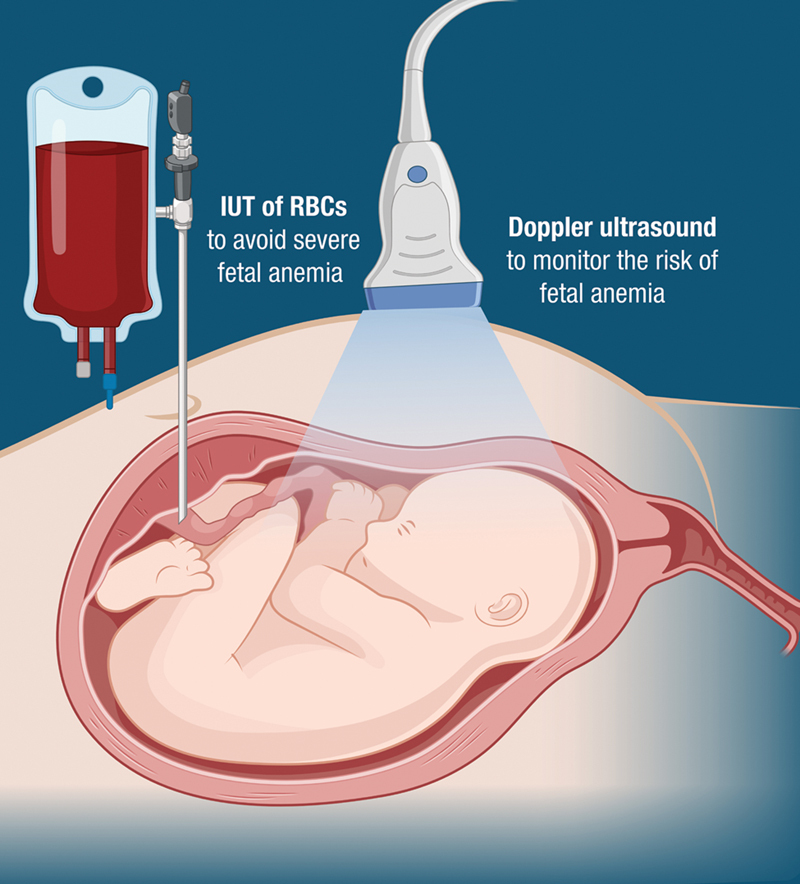
Current management of pregnancy at risk of severe HDFN. HDFN, hemolytic disease of the fetus and newborn.

When pregnancies are likely to develop severe HDFN, current treatment involves noninvasive monitoring with Doppler ultrasound of blood flow in the brain for signs of fetal anemia. If these signs are identified, fetal anemia is confirmed by taking a fetal blood sample (cordocentesis). If anemia is confirmed, the fetus is immediately given an IUT of RBCs to prevent severe fetal anemia, fetal hydrops, and fetal death. IUT is an invasive procedure with a risk of complications such as preterm birth, fetal loss, and fetal–maternal bleeding, which can further increase production of maternal alloantibodies ( Fig. 3 ).

In certain cases and at some clinics, intravenous immunoglobulin (IVIG), plasmapheresis, or a combination of both may be given weekly, starting early in the second trimester, to decrease or delay onset of fetal anemia in severe HDFN. IVIG contains a mixture of many normal IgG antibodies made from the pooled blood plasma from thousands of healthy donors. Plasmapheresis, also known as therapeutic plasma exchange, is a procedure that involves the removal of proteins, including IgG antibodies, from the patient's blood. Both treatments may lower anti-RBC alloantibodies in maternal circulation. These treatments are typically used in pregnancies at high risk of very severe HDFN (early-onset severe HDFN in which fetal anemia developed at 24 weeks of pregnancy or earlier in the previous pregnancy) and have been shown to delay or prevent serious effects of early-onset severe HDFN in some studies. However, both treatments require frequent and long infusions or procedures and can have significant side effects. They are also expensive and may not be covered by insurance since they have not been tested in more rigorous clinical trials and are not approved to treat HDFN.

### What Is Nipocalimab?

Nipocalimab is a weekly intravenous infusion that is being tested in clinical trials for the prevention of severe HDFN. In a small phase 2 clinical trial in pregnant individuals at risk of very severe HDFN, weekly infusions of nipocalimab during the second and third trimesters showed beneficial effects in preventing or delaying fetal anemia and tolerable side effects, which supported its further investigation. The AZALEA study is now testing nipocalimab treatment in pregnant individuals at risk of developing severe HDFN. This is the only therapy currently being tested in a rigorous phase 3 study for the prevention of severe HDFN.

**Fig. 4:**
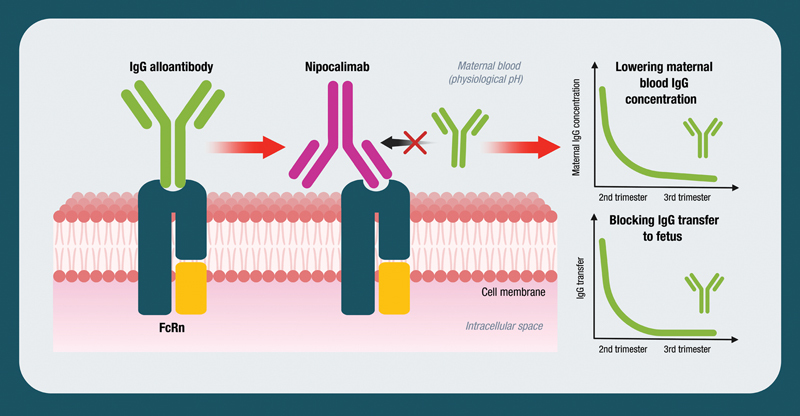
Nipocalimab's mechanism of action.

Nipocalimab works by blocking the neonatal Fc receptor (FcRn). FcRn is considered as the only transporter that can carry IgG, including anti-RBC alloantibodies, across the placenta from maternal to fetal circulation. FcRn also keeps maternal IgG stable at high levels. By blocking FcRn, nipocalimab aims to prevent fetal and neonatal anemia by decreasing the amount of maternal IgG, including anti-RBC alloantibodies, that can enter the fetus and to reduce the level of maternal IgG, including anti-RBC alloantibodies, available to be transferred to the fetus ( Fig. 4 ).

### How Did Nipocalimab Perform in the Phase 2 Clinical Trial for Hemolytic Disease of the Fetus and Newborn?

The phase 2, open-label trial called UNITY included 13 pregnant participants who were at high risk for early-onset severe HDFN. In the UNITY trial, nipocalimab delayed or prevented fetal anemia and IUTs, with a better outcome than was seen in a group of pregnant individuals with the same characteristics from past datasets (known as “historical controls”) that were used for comparison. Benefits in other clinical outcomes were seen in the study pregnancies receiving nipocalimab compared with outcomes in the participants' most recent qualifying pregnancies (recent previous pregnancy from the same participant that was affected by early-onset severe HDFN). For example, in the study pregnancies, fewer participants had IUTs, the median gestational age (or week of pregnancy) was later for the timing of first IUT and delivery, and no fetal hydrops developed. In 46% of pregnancies treated with nipocalimab, none of the maternal participants or their infants needed any type of transfusion (i.e., IUT, exchange transfusion, simple transfusion). Nipocalimab also performed better than IVIG treatment combined with standard practices or standard practices alone (Doppler ultrasound for fetal anemia and IUT[s]) in pregnancies at risk of early-onset severe HDFN as reported by several retrospective studies (a type of study using previously collected data from patient records).

**Fig. 5:**
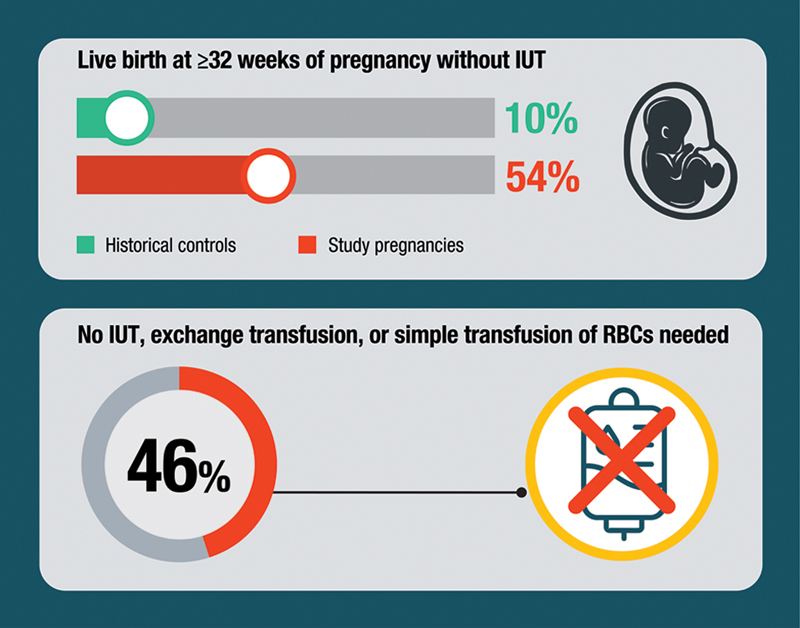
Key clinical outcomes of nipocalimab in the phase 2 UNITY study.

The most frequently reported side effects were those expected with HDFN, pregnancy, or preterm birth and were not necessarily related to the drug being tested. Because IgG is a part of the immune system that fights infection and nipocalimab works by lowering maternal IgG in circulation and blocking IgG transfer to the fetus during pregnancy, infections were a side effect of interest. However, infections in maternal participants or infants were in line with those that typically occur during pregnancy and the neonatal and infancy periods. Overall, the safety and efficacy results in the phase 2 study support further evaluation of nipocalimab in a phase 3 trial, like AZALEA ( Fig. 5 ).

### Why Is the Phase 3 AZALEA Study Being Conducted?

Before a therapy can be approved for use, researchers must study how it works by conducting large clinical studies (known as phase 3 trials). These studies typically compare the new therapy with a placebo to see whether the observed effects are actually caused by the therapy (known as “placebo-controlled” studies). The studies are also “randomized,” which means that a computer program is used to randomly assign the treatment (i.e., drug being tested or placebo) that participants receive. The AZALEA study is a phase 3, global, multicenter, randomized, placebo-controlled study that will test the long-term safety and efficacy of nipocalimab in a larger group of pregnant individuals at risk of severe HDFN before receiving regulatory approval.

### Where Will the Study Take Place, and Who Will Take Part in the Study?

**Fig. 6:**
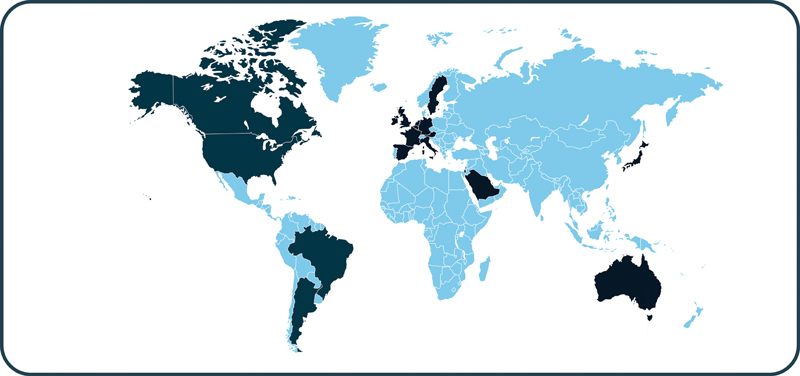
Locations of participating centers in the AZALEA study. (Countries of participating centers are defined in dark blue. For more information on the AZALEA study sites, please visit *https://clinicaltrials.gov/study/NCT05912517* )

**Fig. 7:**
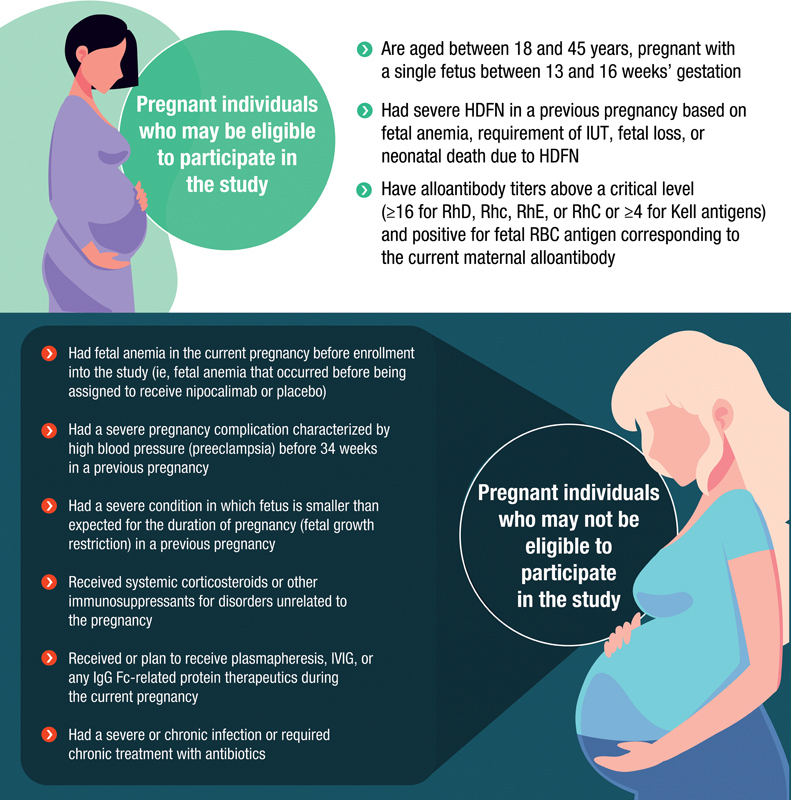
Selected eligibility criteria. (Complete eligibility criteria can be found at the original article and the AZALEA study by visiting *https://clinicaltrials.gov/study/NCT05912517* )

The study is enrolling approximately 120 pregnant individuals who are at risk for severe HDFN based on pregnancy history. Because severe HDFN is a rare condition, participants will be identified at many centers worldwide to ensure enough data are collected. Approximately 60 global centers specializing in maternal–fetal medicine and the treatment of HDFN will be involved in the trial ( Figs. 6 and 7 ).

### How Will the Study Be Performed?

**Fig. 8:**
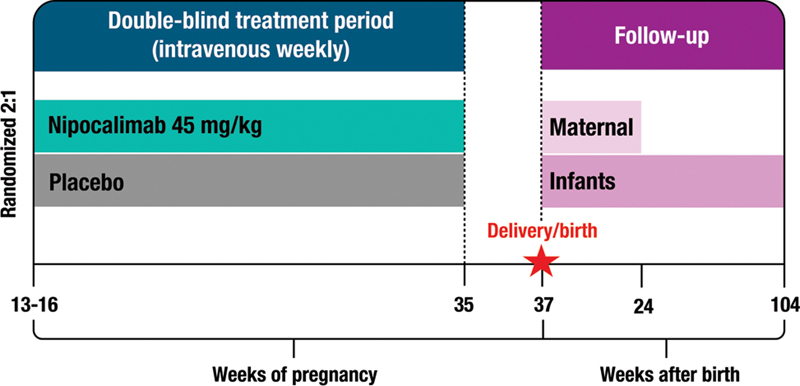
Study design.

In this study, approximately 80 participants will receive nipocalimab at a dose of 45 mg/kg and 40 participants will receive placebo along with weekly monitoring for fetal anemia, as is usual practice for severe HDFN. Both groups will receive intravenous infusions of their assigned treatment weekly from the time they enter the study (between 13 and 16 weeks' gestation) until 35 weeks of pregnancy. The AZALEA trial uses a “double-blind” design to protect against study bias. This means that neither the researchers nor the participants know if the participant is receiving nipocalimab or placebo, and the researchers only find out once the trial is finished. During the double-blind treatment period, participants will be assessed for fetal anemia each week until planned delivery at 37 to 38 weeks of pregnancy. If fetal anemia does develop, participants will be given one or more IUTs as needed and nipocalimab or placebo treatments will be stopped ( Fig. 8 ).

### How Do Researchers Measure the Efficacy of Nipocalimab?

The researchers will compare the proportion of pregnant participants whose pregnancy does not result in fetal loss, IUT, fetal hydrops, or neonatal death (occurs from birth through 4 weeks of age or from the first day of the maternal participant's last menstrual period to 41 weeks of the current date, whichever is later) for those receiving nipocalimab compared with placebo (known as the “primary endpoint” for the AZALEA study). The primary endpoint is the most important result to understand if nipocalimab treatment works in pregnant participants at risk for severe HDFN, which is how the researchers determine the efficacy of nipocalimab.

**Fig. 9:**
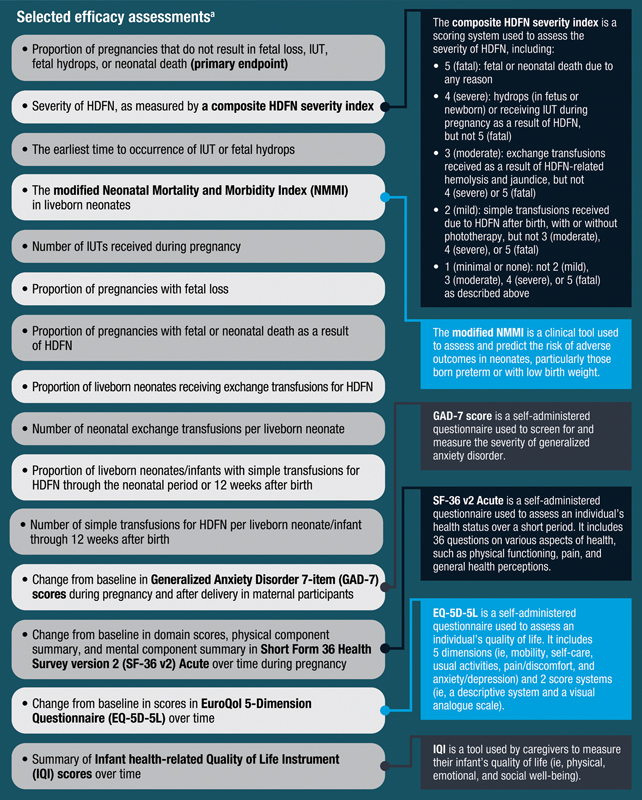
Efficacy and safety assessments. (Complete efficacy assessments can be found in the original article and at *https://clinicaltrials.gov/study/NCT05912517* )

The researchers will also measure other clinical outcomes and ask participants and caregivers to complete questionnaires related to their health and experience with treatment. Fig. 9 shows key assessments related to efficacy that will be measured during pregnancy and after delivery in maternal participants and infants.

### How Do Researchers Monitor the Safety of Nipocalimab?

Maternal participants and liveborn infants will be monitored for any side effects, which may or may not have been caused by their assigned treatment. Researchers will record side effects that occur during the pregnancy and for 6 months after delivery in maternal participants and for the first 2 years of life in infants. Certain side effects that could be related to the way nipocalimab works in the body will also be important to record; these could include infections, low levels of albumin (a protein in the blood) in pregnant participants, and low levels of IgG (<3 g/L) in infants. In addition, infant development will also be evaluated using a standardized tool called the Bayley Scales of Infant and Toddler Development.

### What Impact Will the Results of the AZALEA Study Have?

The AZALEA trial is the first global, multicenter study of its kind that tests the safety and effectiveness of nipocalimab in delaying or preventing fetal anemia and in reducing the need for IUT in pregnant individuals at risk for severe HDFN and the need for postnatal care in their affected infants. The results of this study will provide data necessary for the approval of nipocalimab for use in at-risk HDFN pregnancies. Additionally, these results may enable future clinical research with nipocalimab in other immune-related diseases during pregnancy. They may also provide important insights into the disease biology of HDFN, leading to further improvements in disease management and broader awareness of patients' experiences and needs relating to HDFN.

### Where Can Readers Find More Information on This Study?

You can read the original article here:

Komatsu Y, Verweij EJTJ, Tiblad E, et al. Design of a phase 3, global, multicenter, randomized, placebo-controlled, double-blind study of nipocalimab in pregnancies at risk for severe hemolytic disease of the fetus and newborn. *Am J Perinatol* . Published online September 17, 2024. doi:10.1055/a-2404-8089

*https://www.thieme-connect.com/products/ejournals/html/10.1055/a-2404-8089*

The full name of the AZALEA study is: A Study of Nipocalimab in Pregnancies at Risk for Severe Hemolytic Disease of the Fetus and Newborn. The AZALEA trial started on December 20, 2023, and is expected to end in 2029.

You can read more about the AZALEA study by visiting *https://clinicaltrials.gov/study/NCT05912517* . An infographic summary of this article is available in the Supplementary Material S1 . If you are interested in participating in the study or have questions about the study or nipocalimab, please visit *https://azaleatrial.com/* . More information on clinical studies in general can be found at *https://www.clinicaltrials.gov/ct2/about-studies/learn* .

