# Supplementary material for: Plain Language Summary of Publication: Design of the Phase 3 AZALEA Trial of Nipocalimab in Severe Hemolytic Disease of the Fetus and Newborn
Source: Am J Perinatol. 2025 Mar 6;42(7):955–61. doi: 10.1055/a-2529-4150 (PMC12020508; doi:10.1055/a-2529-4150)
Supplement: Supplementary file 1 — Supplementary Material [file 10-1055-a-2529-4150-s24dec0778.pdf]

Nipocalimab is being evaluated in a phase 3 randomized, controlled trial for pregnancies at risk for severe HDFN.

What is hemolytic disease of the fetus and newborn (HDFN)?

HDFN is caused by a mismatch between the antigens on red blood cells (RBCs) of a pregnant individual and their developing fetus. As a result, the pregnant individual's immune system produces pathogenic immunoglobulin G (IgG) antibodies or alloantibodies that attack the fetal RBCs, causing fetal or neonatal anemia to develop. Antigens on RBCs that commonly cause severe HDFN include Rhesus D (RhD) and Kell.

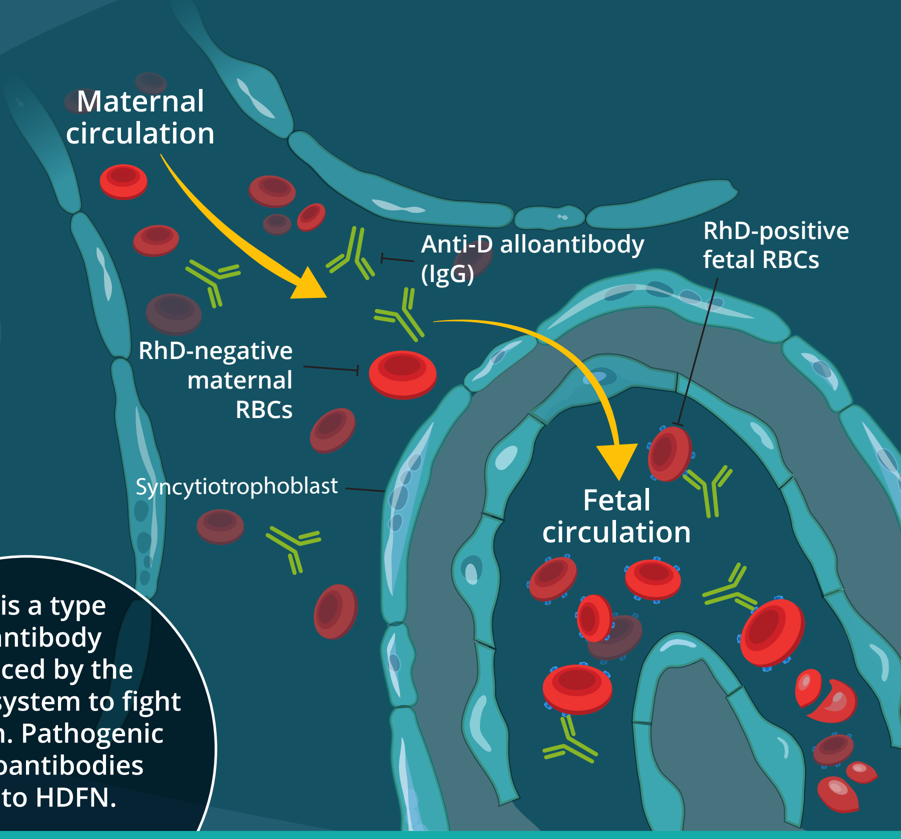

IgG is a type of antibody produced by the immune system to fight infection. Pathogenic IgG alloantibodies lead to HDFN.

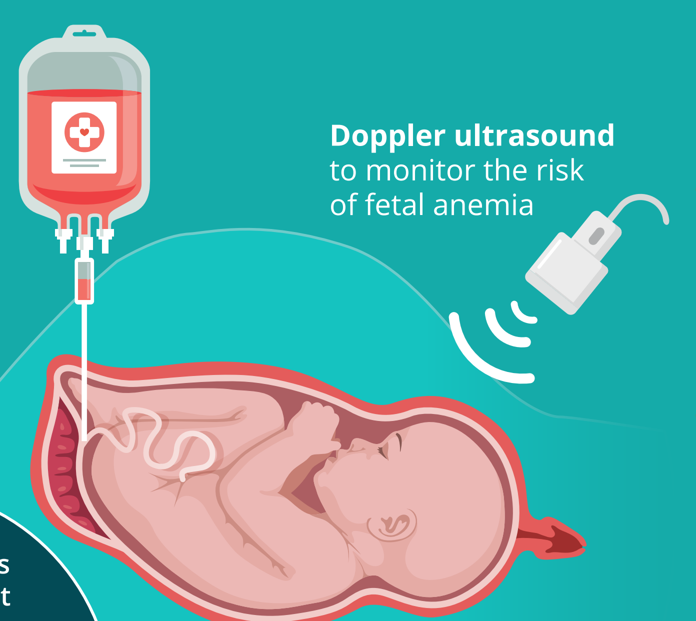

Cordocentesis is a procedure that is performed to obtain a sample of fetal blood for confirmation of fetal anemia.

How is HDFN treated now?

- When pregnancies are at high risk for HDFN, fetal anemia is monitored with **Doppler ultrasound**. If fetal anemia is present, an **intrauterine transfusion (IUT)** of RBCs is administered
- However, the IUT is an **invasive intervention** that is associated with **procedural complications**, including an increased risk of preterm birth or fetal loss
- After birth, affected neonates or infants may require **simple RBC transfusions** or, in severe cases, **exchange transfusions**

How does nipocalimab work?

- Nipocalimab is **currently in clinical development for severe HDFN**
- Nipocalimab works by **binding to the neonatal Fc receptor (FcRn)**
- **FcRn** is the only transporter for IgG in the placenta, responsible for transferring IgG antibodies to the fetus and keeping maternal IgG levels stable

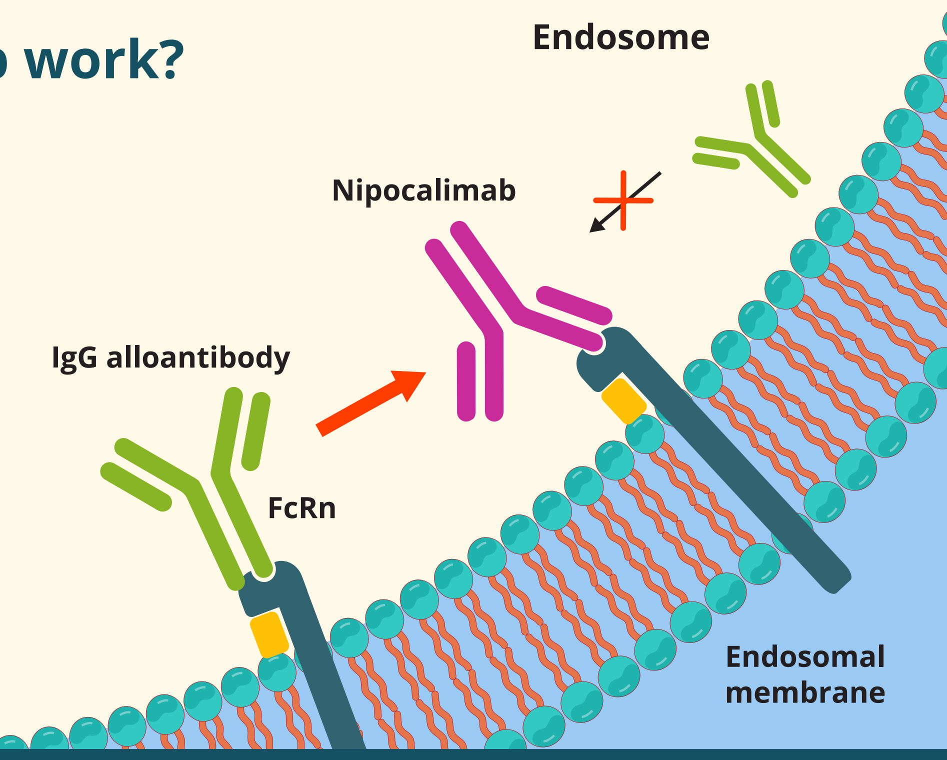

How did nipocalimab perform in the phase 2 clinical trial for HDFN?

- In the phase 2 open-label study, nipocalimab **delayed or prevented fetal anemia and IUTs** in pregnancies at high risk for early-onset severe HDFN as compared with historical controls

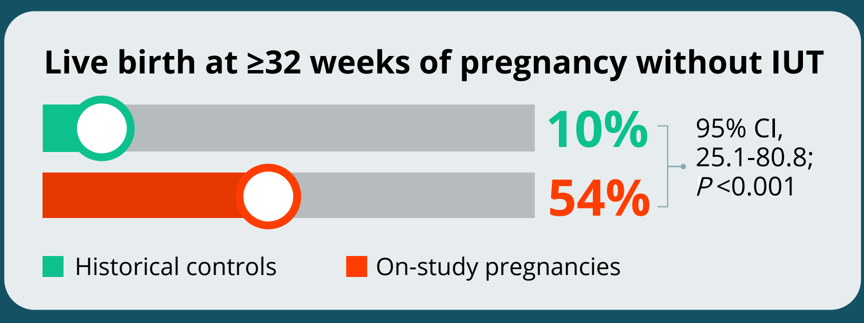

- The most frequently reported **adverse events** included those **common in pregnancy and HDFN**
- In **46% of pregnancies** treated with nipocalimab, **neither maternal participants nor their infants required any transfusions**

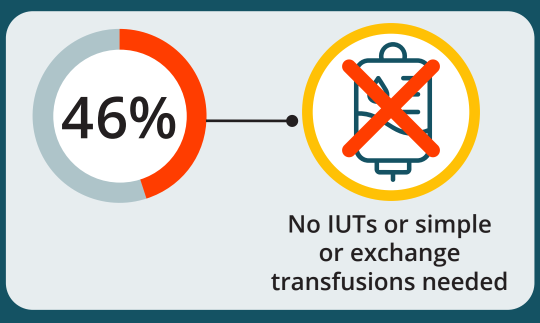

- The safety and efficacy results **support further evaluation** of nipocalimab in a pivotal phase 3 trial

The AZALEA study is the first placebo-controlled, double-blind, randomized, global, multicenter phase 3 trial designed to evaluate the safety and efficacy of nipocalimab in at-risk HDFN pregnancies.

Study population

Pregnant individuals aged 18 to 45 years who:

- Are carrying 1 developing fetus between 13 and 16 weeks' gestation
- Had **severe HDFN in a previous pregnancy** based on fetal anemia, requiring ≥1 IUT, fetal loss, or neonatal death due to HDFN
- Have alloantibody titers and an antigen-positive fetus in the current pregnancy

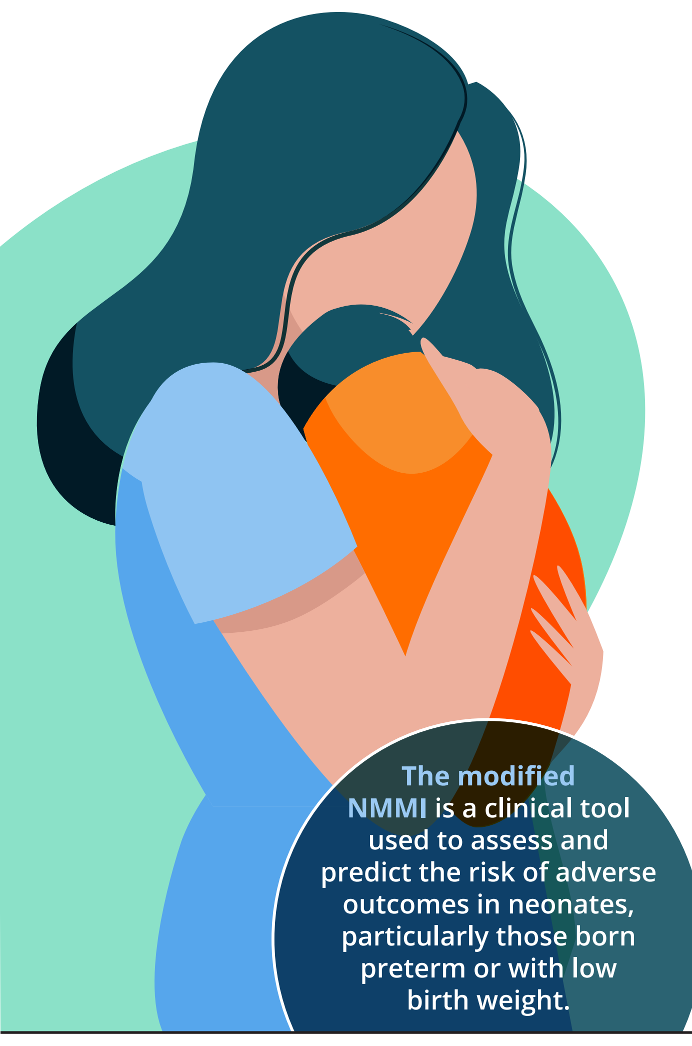

The modified NMMI is a clinical tool used to assess and predict the risk of adverse outcomes in neonates, particularly those born preterm or with low birth weight.

Study design

- Pregnant participants (N ≈ 120) are randomized 2:1 to receive intravenous **nipocalimab or placebo weekly** from 13-16 to 35 weeks' gestation
- During the treatment period, participants **receive weekly monitoring** for fetal anemia until planned delivery at 37 to 38 weeks' gestation. If fetal anemia is diagnosed, an IUT will be administered
- After delivery, **maternal and infant follow-up** will be **6 months and 2 years,** respectively

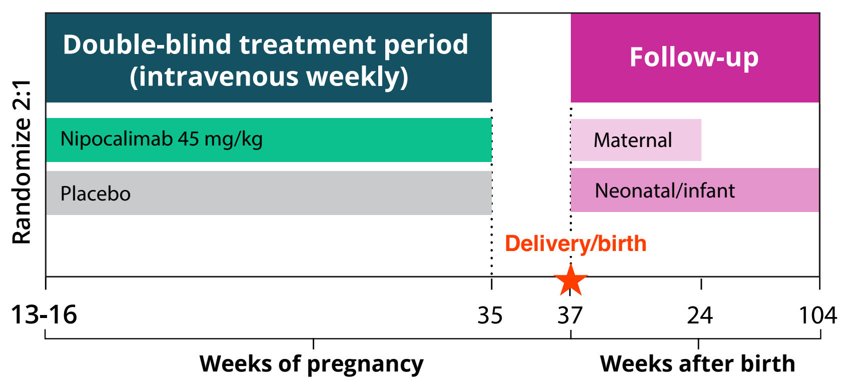

Study assessments

Primary endpoint:

- The proportion of **pregnancies that do not result in fetal loss, IUT, hydrops fetalis, or neonatal death**

Hydrops fetalis (or fetal hydrops) is a serious condition in which abnormal amounts of fluid buildup in ≥2 body areas of a fetus.

Key secondary endpoints:

- Severity of HDFN, as measured by a composite HDFN severity index
- The earliest time to occurrence of IUT or hydrops fetalis
- The modified neonatal mortality and morbidity index (NMMI) in liveborn neonates
- The number of IUTs received

Other assessments:

- Safety outcomes: maternal and infant adverse events
- Pharmacokinetics, pharmacodynamics, and immunogenicity of nipocalimab

Komatsu Y, et al. Design of a phase 3, global, multicenter, randomized, placebo-controlled, double-blind study of nipocalimab in pregnancies at risk for severe hemolytic disease of the fetus and newborn. *Am J Perinatol*. Published online September 17, 2024. doi:10.1055/a-2404-8089
